# Computational fluid dynamics simulate optimal design of segmental arteries reattachment: Influence of blood flow stagnation

**DOI:** 10.1016/j.xjon.2023.07.008

**Published:** 2023-07-22

**Authors:** Yuki Ikeno, Yoshishige Takayama, Michael L. Williams, Yujiro Kawaniashi, Paul Jansz

**Affiliations:** a Department of Cardiothoracic Surgery, St Vincent Hospital Sydney, Sydney, New South Wales, Australia; b Division of Simcenter Support, Department of CCM, Siemens K.K., Tokyo, Japan

**Keywords:** basic science, computational fluid dynamics, spinal cord injury, thoracoabdominal aortic aneurysm repair

## Abstract

**Objectives:**

This study aimed to simulate blood flow stagnation using computational fluid dynamics and to clarify the optimal design of segmental artery reattachment for thoracoabdominal aortic repair.

**Methods:**

Blood flow stagnation, defined by low-velocity volume or area of the segmental artery, was simulated by a 3-dimensional model emulating the systolic phase. Four groups were evaluated: direct anastomosis, graft interposition, loop-graft, and end graft. Based on contemporary clinical studies, direct anastomosis can provide a superior patency rate than other reattachment methods. We hypothesized that stagnation of the blood flow is negatively associated with patency rates. Over time, velocity changes were evaluated.

**Results:**

The direct anastomosis method led to the least blood flow stagnation, whilst the end-graft reattachment method resulted in worse blood flow stagnation. The loop-graft method was comparatively during late systole, which was also influenced by configuration of the side branch. Graft interposition using 20 mm showed a low-velocity area in the distal part of the side graft. When comparing length and diameter of an interposed graft, shorter and smaller branches resulted in less blood flow stagnation.

**Conclusions:**

In our simulation, direct anastomosis of the segmental artery resulted in the most efficient design in terms of blood flow stagnation. A shorter (<20 mm) and smaller (<10 mm) branch should be used for graft interposition. Loop-graft is an attractive alternative to direct anastomosis; however, its blood flow pattern can be influenced.

## Graphical abstract

**Figure:**
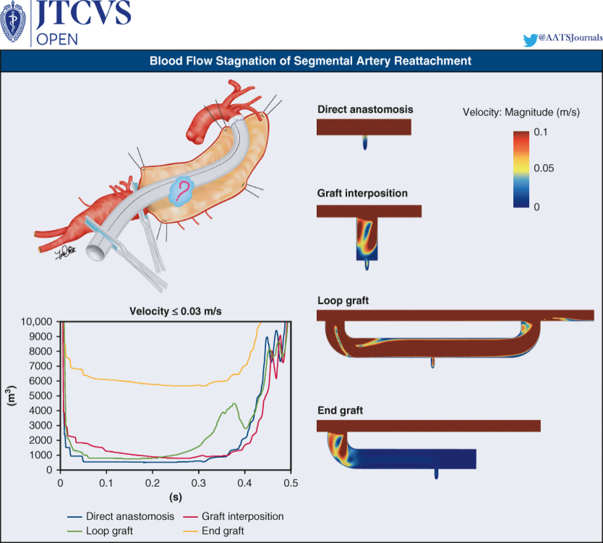

**Figure:**
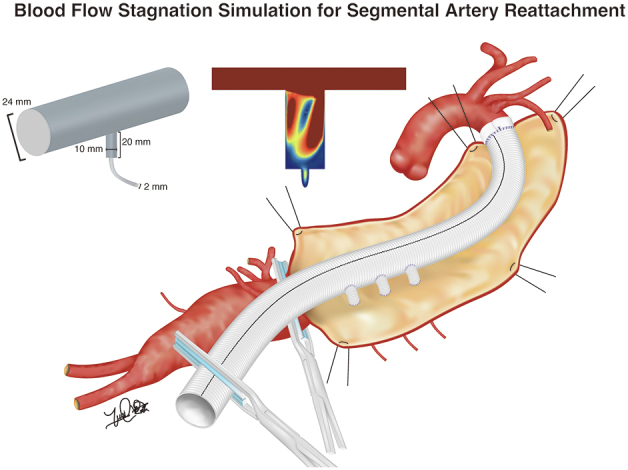
Blood flow stagnation simulation for segmental artery reattachment.

**Central Message**

Computational fluid dynamics can simulate blood flow stagnation, which is a promising indicator to evaluate optimal design of segmental artery reattachment.

**Perspective**

The model-based development approach using computational fluid dynamics can be applicable to pursuing superior method of segmental artery reattachment. Simulation of blood flow stagnation is consistent with current clinical outcomes and explains the paucity of patency rates of each design. The computational fluid dynamics model can be a valuable component of future preoperative assessments.

Spinal cord injury (SCI) remains among the most serious complications of thoracoabdominal aortic aneurysm (TAAA) intervention, with paraplegia occurring at a rate of 2.9% to 16.0%, even in experienced centers.^1^^,^^2^ Although there is no single measure that can prevent paraplegia,^3^ spinal cord protection is classified into 2 major strategies: optimizing spinal cord perfusion and enhancing ischemic tolerance.^4^ Because spinal cord circulation depends on collateral networks consisting of segmental arteries, including the artery of Adamkiewicz, intercostal artery reattachment has been proposed to attenuate rates of SCI.^5^, ^6^, ^7^ There have been several options reported to reconstruct segmental arteries: direct anastomosis,^1^ graft interposition,^8^ loop-graft technique,^9^ and end-graft technique, with ranging patency of 31% to 91%.^10^^,^^11^ It is hypothesized that higher patency rates of reattached segmental arteries could prevent ischemic injury^12^; however, the optimal reattachment design for improved patency remains controversial.

Model-based development (MBD) is a method of evaluating product performance through simulation and optimization by representing the entire product on a functional basis before creating a 3-dimensional (3D) model in the early stages of the product design process in the automotive and aerospace industries.^13^ Regarding clinical application of MBD for surgical strategies, computational fluid dynamics (CFD) is an emerging technology, particularly for ischemic heart disease and cerebral artery aneurysms.^14^ Recently, Shiiya and colleagues^15^ reported in their CFD study that persistent helical flow during diastole was beneficial for the patency of reattached intercostal arteries. However, their study investigated the streamline of reattached grafts, which was subjective.

We hypothesize that the blood flow stagnation of a reattached segmental artery influences the patency rate as an objective parameter. The aim of this study was to simulate blood flow stagnation to elucidate the optimal design of reattached segmental arteries for TAAA repair using a CFD model.

## Methods

### Experimental Design

**Figure 1:**
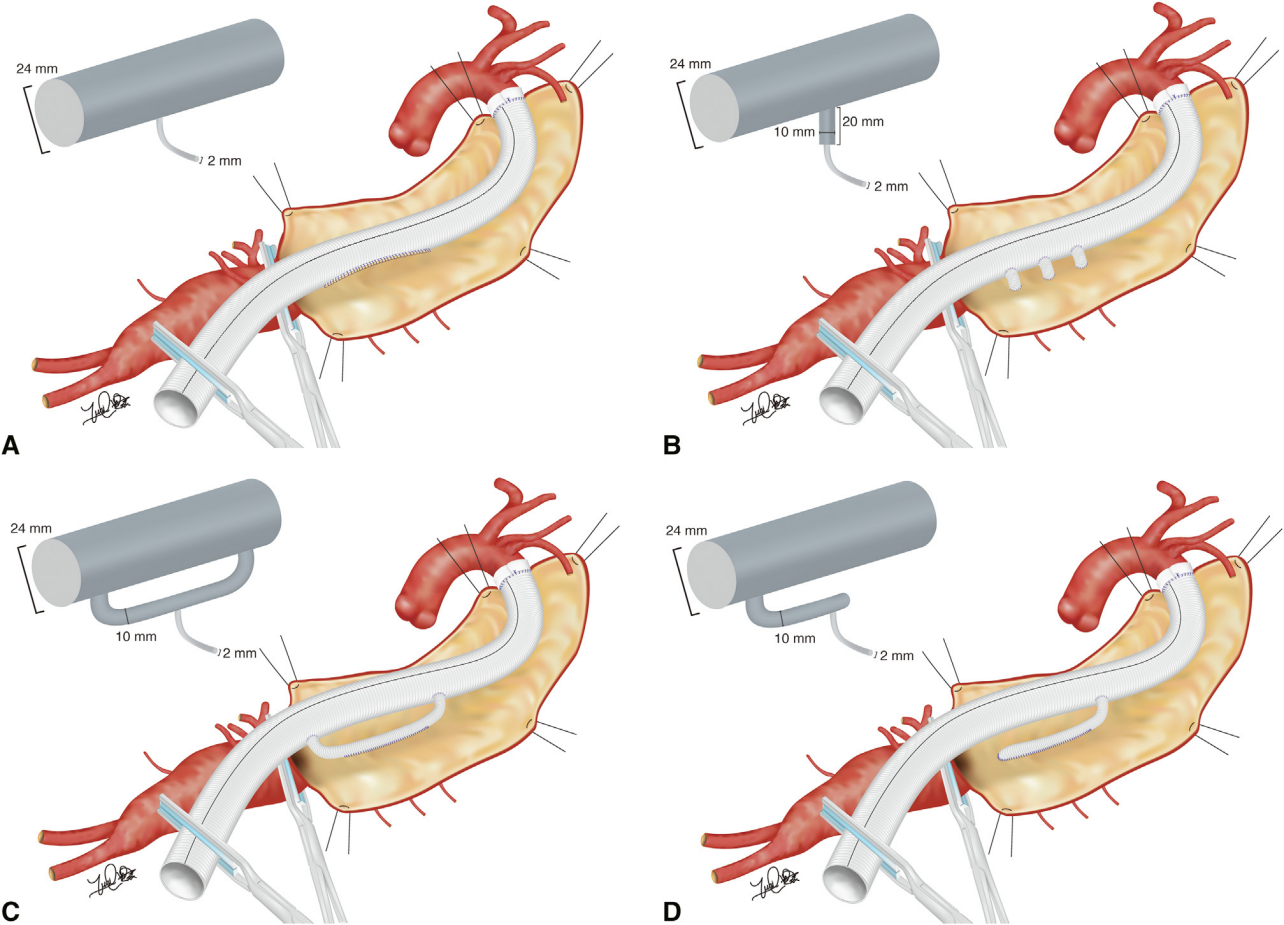
Models of designs of reattached segmental arteries. Diameter of the main trunk of the replaced graft is 24 mm. Diameter of the segmental artery was 2 mm. Side branches for graft interposition, loop graft, and end graft were 10 mm. A, Direct anastomosis. B, Graft interposition. C, Loop graft. D, End graft.

**Figure E1:**
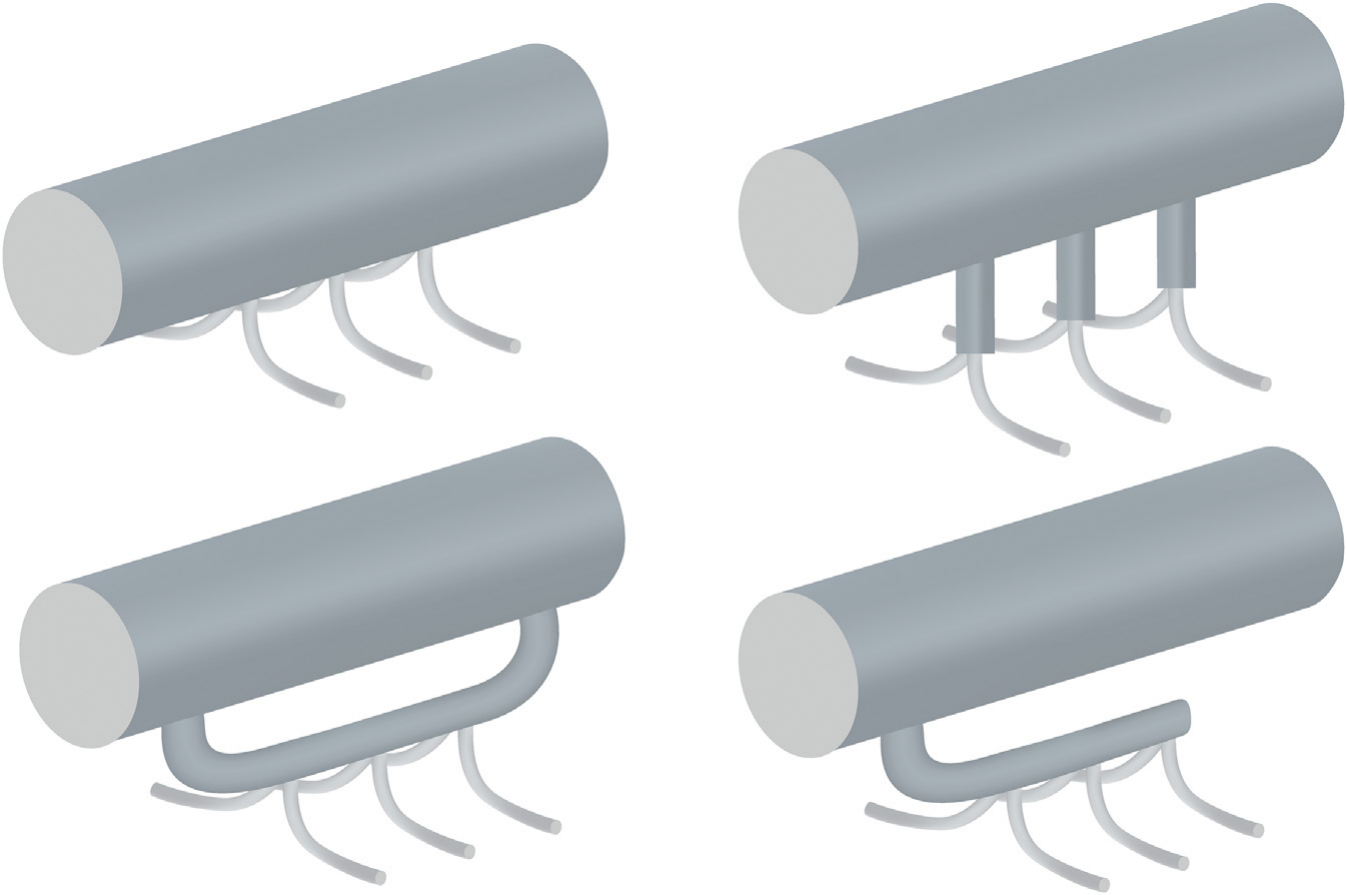
Designs of segmental arteries reattachment with 3 pairs. The distance between each segmental artery was 30 mm.

A 3D CFD model of segmental artery reattachment was developed to reliably evaluate blood flow stagnation. Blood flow stagnation was defined by volume of velocity ≤0.03 m/second simulating the systolic phase (low-velocity volume), which is supposed to increase viscosity of blood.^16^^,^^17^ We also illustrated area of velocity ≤0.03 m/second obtained by plane section at 0.3 seconds of systole to visualize simulated blood flow stagnation (low-velocity area). Four methods of segmental artery reattachment were compared: direct anastomosis, graft interposition, loop-graft, and end graft (Figure 1). The diameter of the main body of the graft, branches, and segmental arteries were based on previous clinical literature.^18^ We evaluated the consistency of blood flow pattern when 3 pairs of segmental arteries were reattached (Figure E1). In terms of graft interposition, we also analyzed different lengths (10 mm, 20 mm, and 30 mm), widths (6 mm, 8 mm, 10 mm, and 12 mm), and configurations (tube and hemisphere) of the interposed graft. For the loop-graft, we also simulated influence of graft configuration on blood flow stagnation.

### CFD Model

#### Governing equations and turbulence model

Simcenter STAR-CCM+ 2022.1 (Siemens Digital Industries Software), a general-purpose thermal-fluid analysis software, was used for the fluid analysis. This analysis assumed incompressible Newtonian fluid. The finite volume method was used to discretize the continuity equation (1) and the Navier-Stokes equations (2),

(1) ∇・v=0

(2) $$\frac{\partial v}{\partial t}+\left(v・\nabla \right)v=-\frac{1}{\rho }\nabla p+\frac{\mu }{\rho }\nabla^{2}v+g$$

where ρ is the density of the fluid, v is the velocity, p is the pressure, and μ is the viscosity, ∇ is vector differential operator, g is the external force acting per fluid mass. The turbulence model was the realizable k-ε model that enables equivalent treatment to low-Reynolds-numbers turbulence models. Second-order accurate upwind and SIMPLE (Semi-Implicit Method for Pressure-Linked Equations) methods were used for discretization and pressure solution, respectively.

#### Mesh

By using the finite volume method, the 3D aortic model is divided into a grid, and each cell or boundary surface has representative values, including velocity and pressure. This calculation uses polyhedral mesh, which has the advantage of having a larger number of faces, which improves analytical stability and reduces the number of cells, thereby reducing the computational load compared with tetrahedral mesh. The mesh size was set to a maximum of 0.75 mm and a minimum of 0.075 mm to sufficiently resolve the flow in the aortic model and set boundary layers to resolve velocity gradient. In this setup, there are 36 nodes on the same circumference of the aortic model. This number is sufficient to represent a circle of a polyethylene terephthalate graft.

#### Calculation conditions

**Figure 2:**
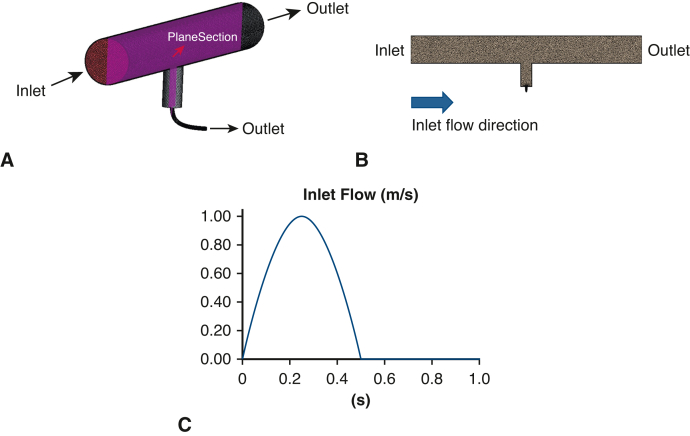
Computational fluid dynamics model. Polyhedral mesh (discretized by finite volume method) based on computer-aided design model simulating abdominal aorta, populated vessels, and intercostal arteries. A, 3-dimentional image. B, plane section. C, Time-series variation of inlet velocity simulating pulsatile flow.

The inlet boundary varies the flow rate and the outlet boundary was given a pressure outlet condition of 120 mm Hg as shown in Figure 2. The wall of aortic model was treated as a rigid wall with a no-slip condition. The blood samples were incompressible Newtonian fluids with density: ρ = 1000 kg/m^3^ and viscosity μ = 0.004 Pa-s.^19^

The difference between compressible and incompressible fluids is whether or not a density change occurs. Compressible fluids that are affected by density changes are generally said to start at Mach numbers = 0.3.

The speed of sound (Mach = 1) of water, which is very close in physical properties to blood, is about 1500 m/second, and the velocity of blood flow is much lower than that. Therefore, the calculation is performed as incompressible.

To simulate a pulsating flow, this calculation was performed with an implicit unsteady analysis, the time step was 1 ms so that the fluid area of the artificial blood vessel, where blood stagnates, has a courant number $\frac{\Delta t}{\Delta x}v$ <1, where Δt is time step, Δx is length of cell. As mentioned above, the courant number is a variable determined by speed and time. In implicit unsteady analysis, it is not required that the courant number is <1, but it was implemented to improve the convergence and accuracy of the calculation. Internal iterations within each time step confirm that the next step is executed when the flow rate (mass balance) or the value of the volume below the specified velocity no longer changes.

## Results

### Low-velocity Volume

**Figure 3:**
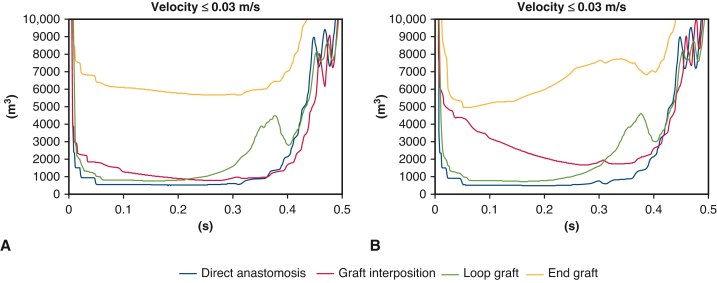
Low-velocity volume in systole for single segmental artery reconstruction (A) and 3 pairs of segmental arteries reconstruction (B). Direct anastomosis resulted in the minimum low-velocity volume (*blue*), whereas end graft showed the maximum low-velocity volume throughout the entire systolic phase (*yellow*). Graft interposition showed slightly larger low-velocity volume than direct anastomosis and loop graft (*red*). The difference of low-velocity volume between graft interposition and direct anastomosis increased when 3 segmental arteries are reconstructed. Loop graft exhibited a velocity pattern that was similar to direct anastomosis, except for fluctuations in velocity during the latter half of the systolic phase (*green*).

The low-velocity volume in systole is summarized in Figure 3, *A*. Direct anastomosis resulted in the least low-velocity volume. When compared with direct anastomosis, end graft showed the largest stagnation of blood flow during the entire systolic phase. Loop-graft showed comparatively less blood flow stagnation over time velocity change; however, it fluctuated particularly during the late systole phase. Graft interposition resulted in significant blood stagnation when compared with direct anastomosis; however, it performed better with less blood stagnation than the end graft reattachment model. These results were consistent when adding the segmental arteries of 3 pairs (Figure 3, *B*). Notably, the low-velocity volume of graft interposition increased compared with single segmental artery reconstruction, whereas direct anastomosis and loop-graft showed similar pattern of low-velocity volume.

### Low-Velocity Area at 0.3 Seconds of Systole

**Figure 4:**
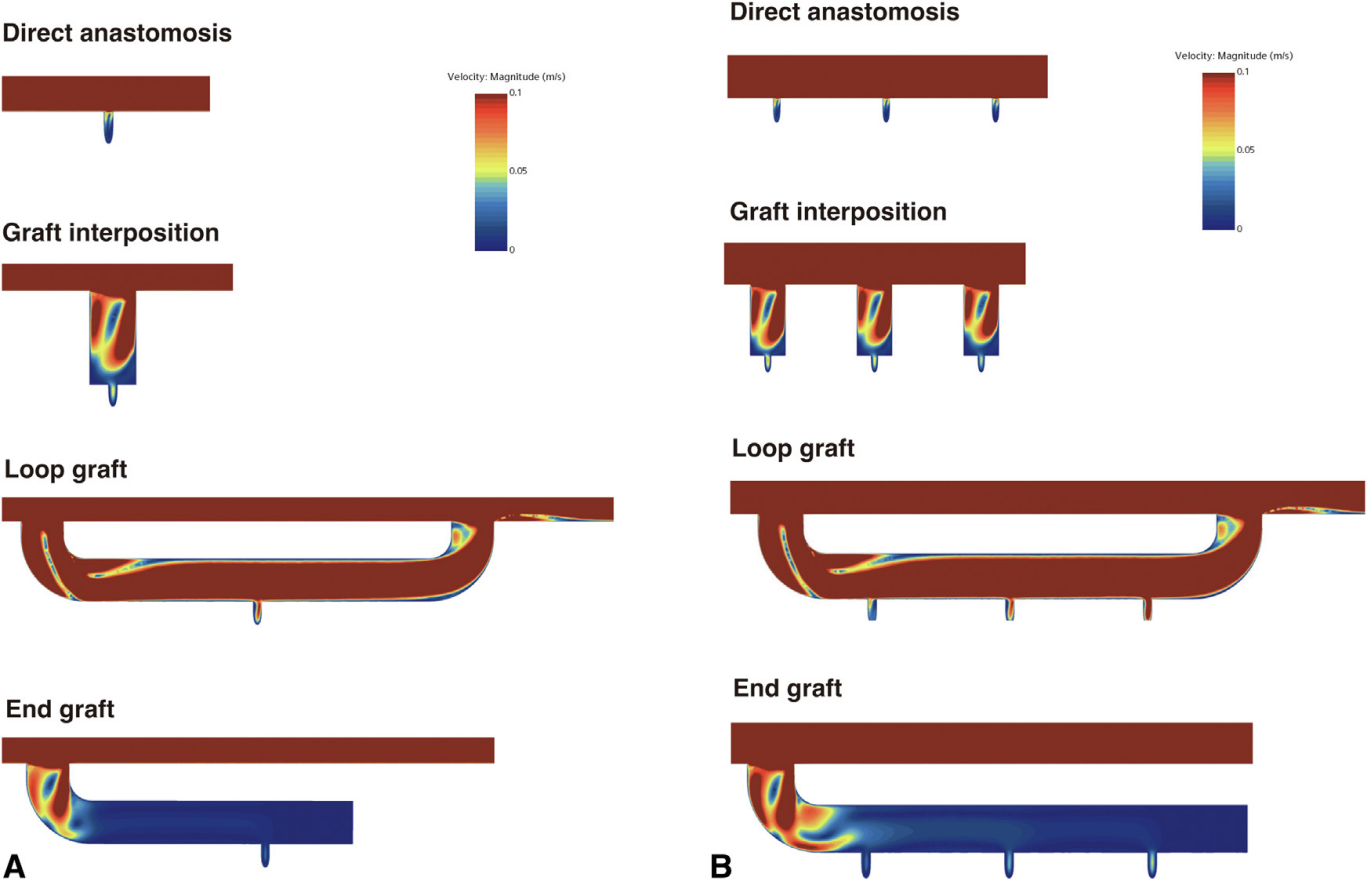
Low-velocity area at 0.3 seconds of systole for single segmental artery reconstruction (A) and 3 pairs of segmental arteries reconstruction (B). There were large low-velocity areas in the end graft. The distal part of the interposed graft also shows a significant low-velocity area.

The low-velocity area at 0.3 seconds of systole is summarized in Figure 4, *A*. A large low-velocity area was identified in the end graft reattachment model. Graft interposition showed significant low-velocity area at the distal end of the branched graft. Similar trends were observed in 3 pairs of reattached segmental arteries (Figure 4, *B*).

The low-velocity area of plane section in the entire systole phase is shown in Video 1. Fluctuation of the low-velocity area during the late systole phase was visualized in the loop graft reattachment model.

### Comparison of Graft Length in Graft Interposition

**Figure 5:**
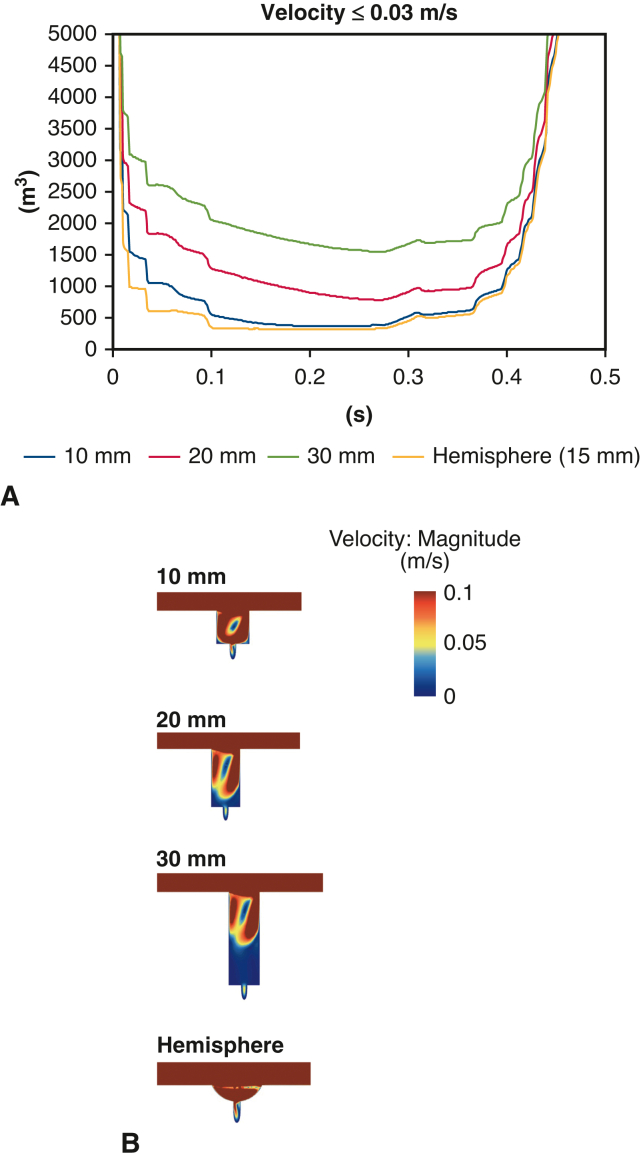
Comparison of graft length and configuration in graft interposition. A, Low-velocity volume. B, Low-velocity area. Shorter interposed graft and hemisphere-shaped graft were associated with less blood flow stagnation.

**Figure E2:**
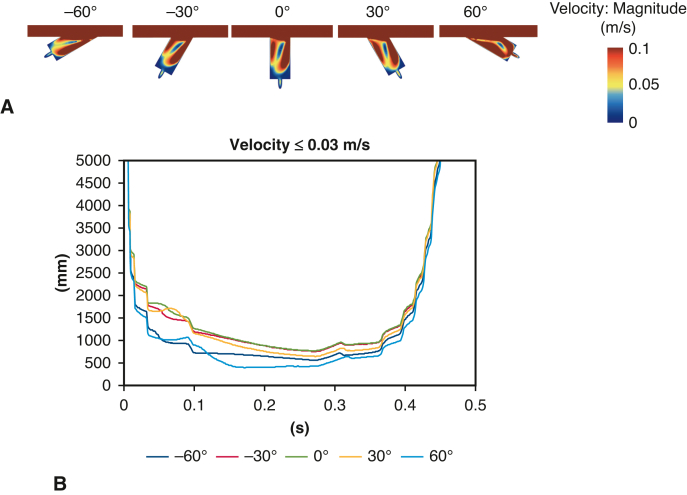
Blood flow stagnation in systole comparing angles of the interposed grafts. A, Low-velocity area at 0.3 seconds. B, Low-velocity volume.

When comparing different graft interposition lengths, the 30-mm graft branch showed significant increase of low-velocity volume compared with the 20-mm graft (Figure 5, *A*). Comparatively, the 10-mm graft resulted in the least low-velocity volume, reflecting the smaller low-velocity area of the periphery of the branch (Figure 5, *B*). The advantage of the shorter branch length was maintained throughout in the entirety of systole. A hemisphere shaped branch of 15 mm diameter showed less low-velocity volume in comparison to any other length of cylindrical branches. We also compared graft interposition and aorta at different angles, resulting in a decrease in the volume of the slow region when the graft and aorta approached parallel (Figure E2, *A* and *B*).

### Comparison of Graft Diameter in Graft Interposition

**Figure 6:**
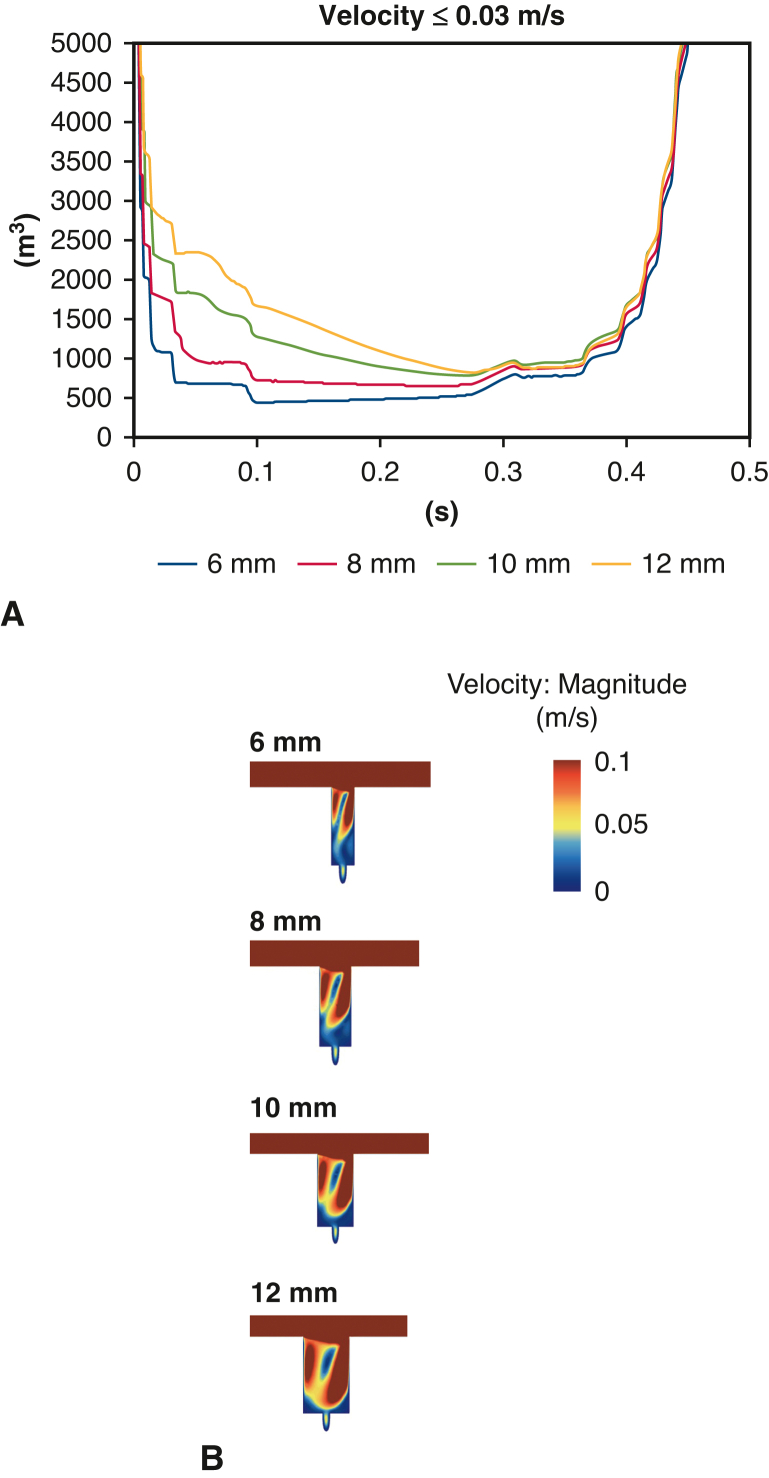
Comparison of graft diameter in graft interposition. A, Low-velocity volume. B, Low-velocity area. Smaller diameter of interposed graft was associated with less blood flow, particularly during early systole.

When comparing graft diameter of an interposed graft, the smaller graft showed less low-velocity volume, particularly during the early systolic phase (Figure 6, *A*). However, the advantage of smaller branch sizes disappeared during late systole. In addition, significant low-velocity areas of the distal of the branch remained even in the graft with a diameter of 6 mm (Figure 6, *B*).

### Influence of the Altered Configuration in the Loop-graft

**Figure 7:**
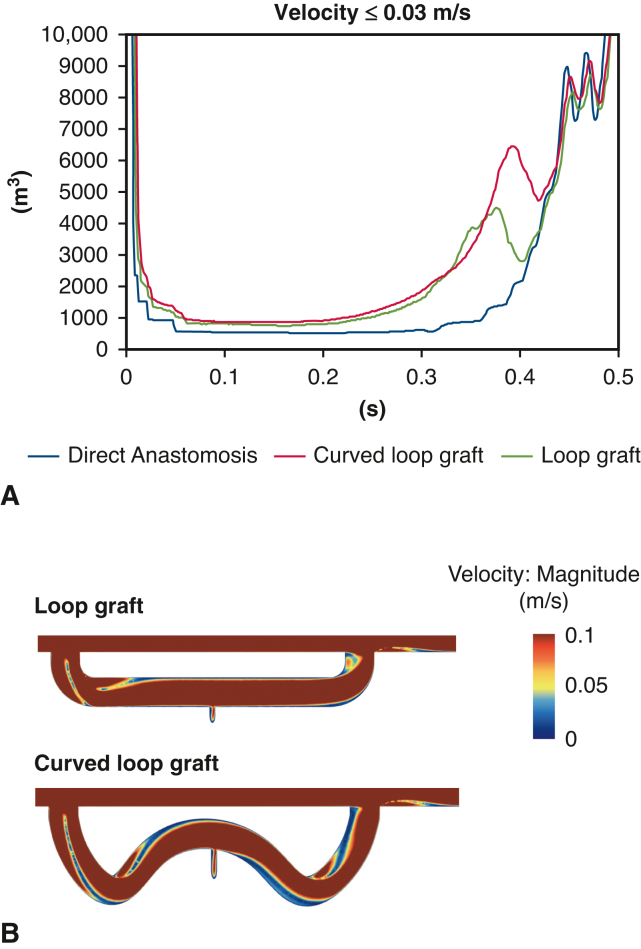
Influence of configuration on loop graft. A, Low-velocity volume. B, Low-velocity area. There was fluctuation in velocity during late systole by curved loop graft.

Although loop-grafts showed comparative low-velocity volume with direct anastomosis, the flow pattern was influenced by varying configurations of the loop-graft (elongated and curved) (Figure 7, *A* and *B*).

## Discussion

**Figure 8:**
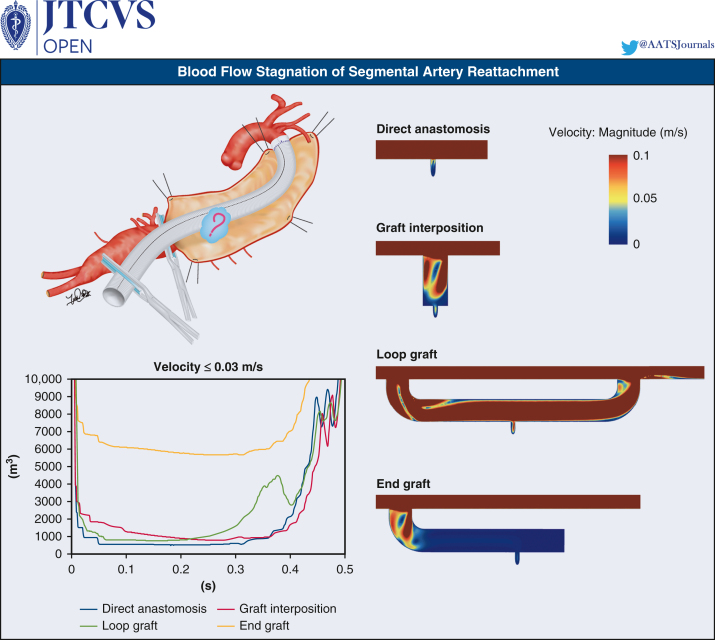
Blood flow stagnation simulation of segmental artery reattachment.

This study demonstrated that a direct anastomosis reattachment model results in the most efficient design to minimize blood flow stagnation. Loop-graft can be a reasonable alternative, although the configuration should be careful not to attenuate its efficiency (Figure 8). For graft interposition, the shorter and smaller grafts were preferable. Hemisphere-shaped grafts might be an intriguing option because low patency is a major concern of graft interposition.^18^

The rationale of segmental artery reattachment and its implication in reducing SCI is an area with current equipoise in the medical literature. Several studies report that sacrifice of intercostal and lumber arteries during TAAA repair is safe on the basis of the acceptable incidence of SCI.^20^^,^^21^ In comparison, Zoli and colleagues^22^ report that the number of sacrificed intercostal arteries was the most powerful predictor of SCI. Afifi and colleagues5 and Estrera and colleagues^23^ also published evidence that intercostal artery reimplantation may be beneficial, even if the artery of Adamkiewicz is not as crucial to spinal cord perfusion. Henmi and colleagues^12^ demonstrated that the patency rate was significantly associated with the incidence of SCI (interposed graft 54.0% and direct anastomosis 77.1%-91.2%). Therefore, when interpreting the above evidence, it appears that segmental artery reattachment is beneficial and further research is required to assess the optimal reattachment model to improve outcomes of SCI in TAAA repair.

Although our CFD simulation were not linked to clinical outcomes, the results of low-velocity volume were consistent with current studies describing patency rates of reattached segmental arteries. Direct anastomosis has been reported as having the highest patency rates of any other model of reattached segmental arteries. However, alternative approaches have also been investigated because there are concerns of surgical bleeding and late aneurysm growth.^11^^,^^12^ For graft interposition, larger low-velocity volume and stagnant blood flow supported relatively low patency, ranging from 31% to 70%.^11^^,^^18^ This range can be explained by the different sizes and lengths of the interposed grafts. Shiiya and colleagues^15^ demonstrated that graft size should be smaller than 10 mm and graft length should be shorter than 25 mm to improve patency. Our simulation showed that graft length was preferable at <20 mm and smaller-diameter grafts were superior to avoid stagnant blood flow because a low-velocity area occurred at the corner of the tubed graft. For this reason, end graft also resulted in the largest low-velocity volume, which supported its worst patency rates in a recent study that compared it to direct anastomosis, graft interposition, and loop-graft reattachment techniques.^11^ Notably, both end graft and graft interposition models had relatively high blood stagnation but showed greater amounts of simulated blood flow to the segmental artery in comparison to the direct anastomosis model (direct anastomosis 0.21 mL, graft interposition 0.27 mL, loop-graft 0.24 mL, and end graft 0.32 mL). Therefore, it appears that blood flow stagnation is the predominant marker to evaluate graft patency over the amount of blood flow. The low-velocity volume of the loop-graft model and its promising patency also support this theory. In addition, when comparing the size of the outflow intercostal vessel, direct anastomosis and loop-grafts showed little change in the volume of the slow region over time, even when the diameter of the segmental artery was changed. This may be due to the fact that the segmental artery and graft or aorta are in the normal direction, which is unlikely to be influenced even if the diameter is slightly increased.

**Figure E3:**
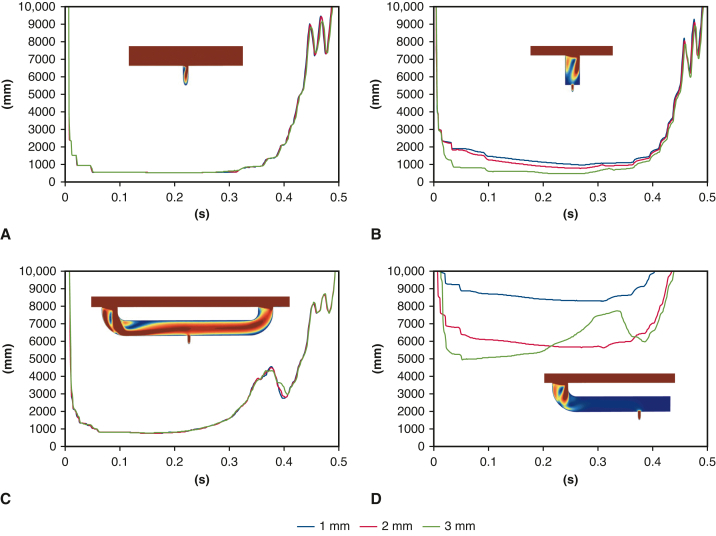
Blood flow stagnation in systole in each reconstruction stratified by the size of the interposed graft (1 mm, 2 mm, and 3 mm).

In the case of graft interposition, the segmental artery and graft are in the same direction, which may have changed the flow pattern, decreasing the slow region at the end of the graft as the diameter of the segmental artery increases. The end graft is slow in many areas to begin with, and it is assumed that the flow pattern is sensitive to differences in the diameter of the segmental artery, and as a result, the slow areas are likely to change as well (Figure E3). However, on the basis of our results, the meticulous design of the loop-graft should be warranted.

The MBD approach, which has been utilized by the aerospace and automotive industries for decades, has now moved in to the medical industry. MBD uses different devices and methodologies to facilitate a systematic and methodological control design, alleviates the need for experimental system calibration, and allows for system integration and requirements testing early in the development stage. A CFD model using both nonpatient-specific and patient-specific imaging can be applicable to pursuing better design of not only segmental artery reattachment, but also any surgical decision making where applicable. We believe that CFD models can be a valuable component of the preoperative assessment to provide superior surgical outcomes in the future.

### Limitations

Although our results are promising, they are not without limitations. First, it was a simulation-based study. Although the results are consistent with recent clinical publications in the literature, clinical data need to be demonstrated. Secondly, our study did not analyze the fragility or elasticity of the aortic wall such as that in a dissected aorta or connective tissue diseases. The indication of direct anastomosis of a segmental artery should be determined in conjunction with other clinical factors. Thirdly, we did not evaluate the influence of remnant aortic tissue due to the equation of the CFD model. In direct anastomosis, island reconstruction and single-cuff technique could not be compared in this study. Fourthly, it is assumed that the segmental artery and its surrounding geometry will change the peripheral vascular resistance and influence the pressure and velocity in the vessel, but this study has not been able to account for this. This is because it is difficult to make precise predictions without capturing the time-series pressure fluctuations in peripheral blood vessels. The results of pressure measurements downstream of intercostal arteries in animal experiments are awaited. Finally, this model did not simulate how collateral networks influenced the thrombosis or patency rates. Patient-specific CFD models and clinical studies assessing the above findings are required.

## Conclusions

Patient-nonspecific CFD was useful to simulate the optimal design for segmental artery reconstruction for TAAA repair. Simulated blood flow stagnation appears to be a promising predictor of patency. Direct anastomosis of the segmental artery resulted in the most efficient design to avoid blood flow stagnation, whereas an end graft reattachment model showed the worst stagnant blood flow. A shorter and smaller tube graft should be used for graft interposition to improve patency. Otherwise, the loop-graft is an attractive alternative to direct anastomosis; however, it can be influenced by its configuration.

### Conflict of Interest Statement

The authors reported no conflicts of interest.

The *Journal* policy requires editors and reviewers to disclose conflicts of interest to decline handling or reviewing manuscripts for which they have a conflict of interest. The editors and reviewers of this article have no conflicts of interest.

## Supplementary Data

Video 1 Changes of low-velocity area in the systole. A, Direct anastomosis. B, Graft interposition. C, Loop-graft. D, End graft. Video available at: https://www.jtcvs.org/article/S2666-2736(23)00193-6/fulltext.
