# Supplementary figures and images for: Computational fluid dynamics simulate optimal design of segmental arteries reattachment: Influence of blood flow stagnation
Source: JTCVS Open. 2023 Jul 22;15:61–71. doi: 10.1016/j.xjon.2023.07.008 (PMC10556939; doi:10.1016/j.xjon.2023.07.008)

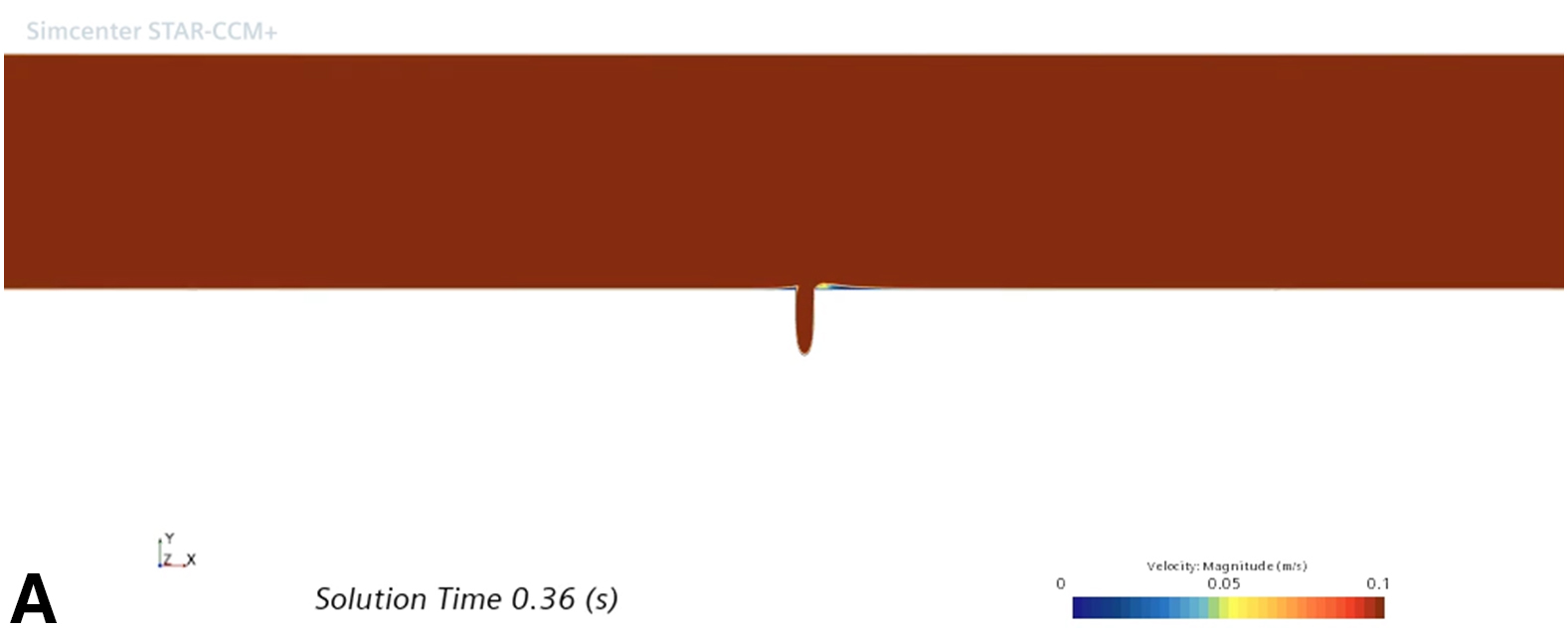

Supplement: Supplementary file 2 [file fx2.jpg]

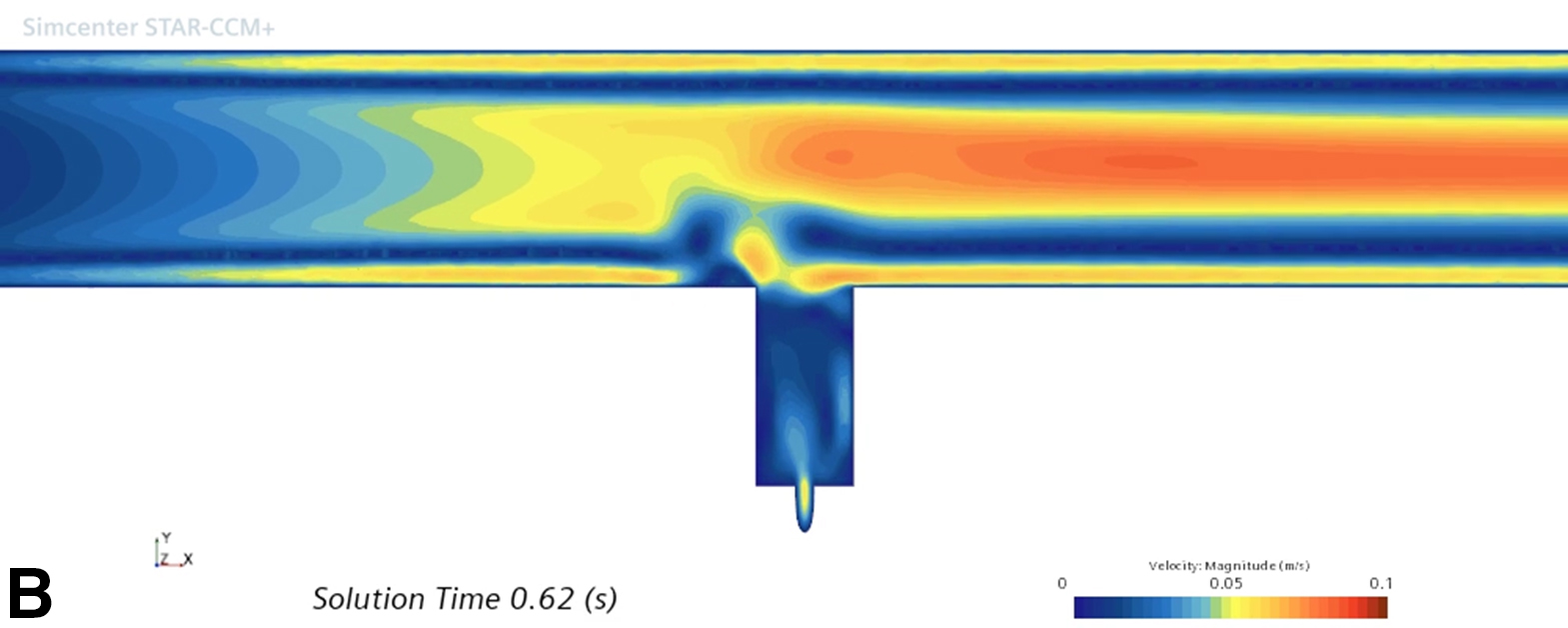

Supplement: Supplementary file 4 [file fx3.jpg]

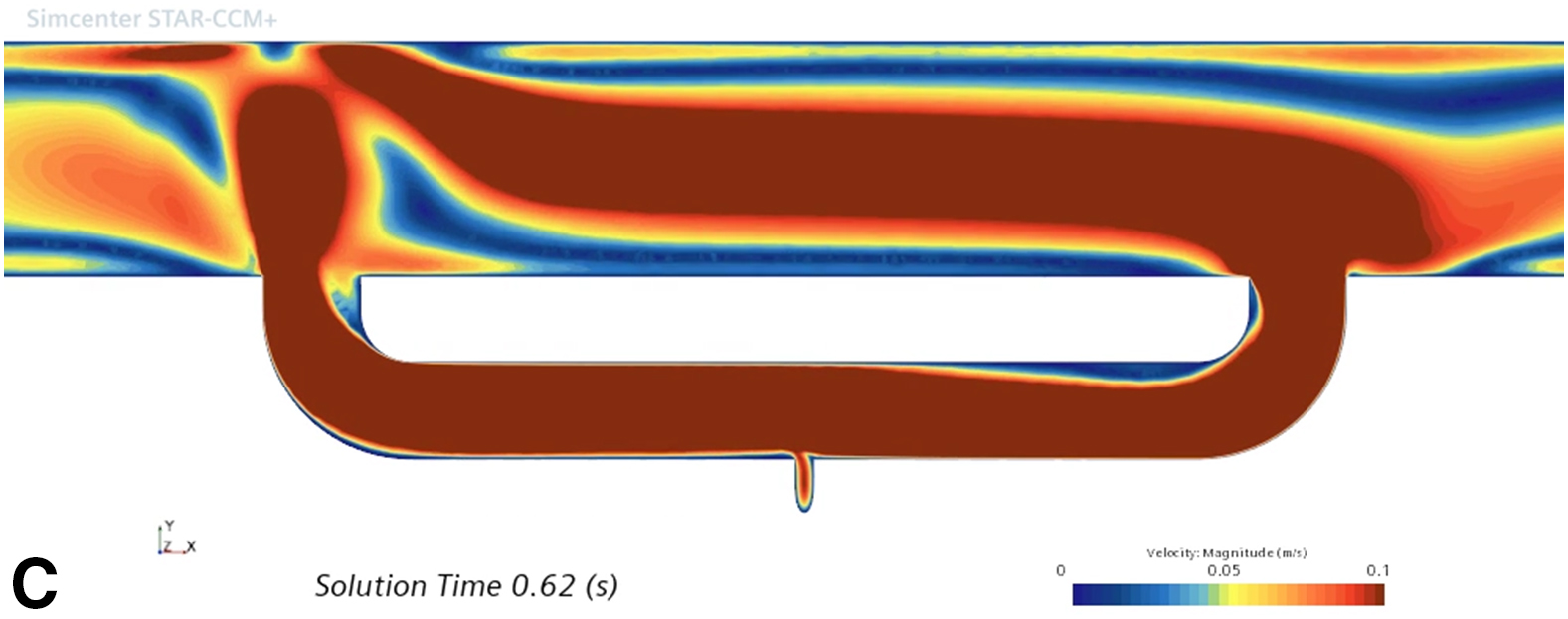

Supplement: Supplementary file 6 [file fx4.jpg]

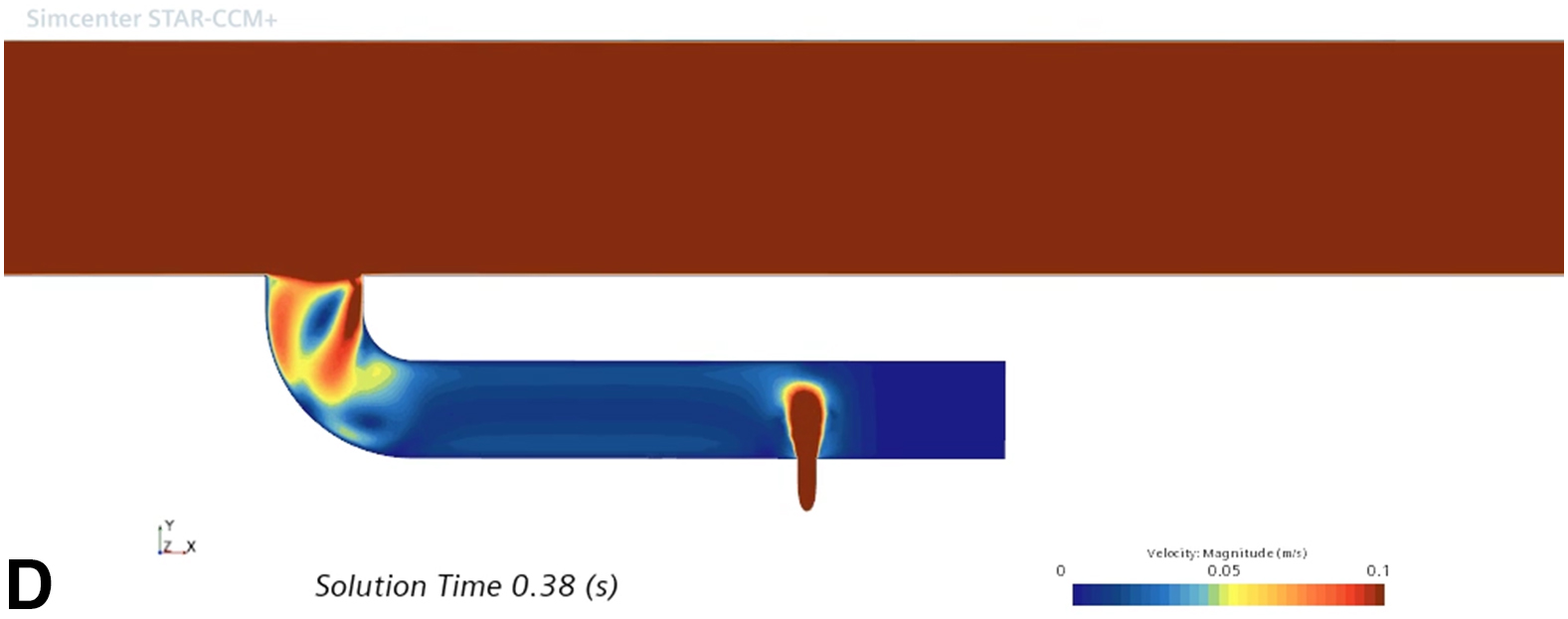

Supplement: Video 1 — Changes of low-velocity area in the systole. A, Direct anastomosis. B, Graft interposition. C, Loop-graft. D, End graft. Video available at: https://www.jtcvs.org/article/S2666-2736(23)00193-6/fulltext. [file fx5.jpg]
